# A Comprehensive Microstructure-Aware Electromigration Modeling Framework; Investigation of the Impact of Trench Dimensions in Damascene Copper Interconnects

**DOI:** 10.3390/nano14221834

**Published:** 2024-11-16

**Authors:** Ahmed Sobhi Saleh, Kristof Croes, Hajdin Ceric, Ingrid De Wolf, Houman Zahedmanesh

**Affiliations:** 1 IMEC, Kapeldreef 75, B-3001 Leuven, Belgium; ahmed.saleh@imec.be (A.S.S.);; 2 Department of Materials Engineering, Faculty of Engineering Sciences, KU Leuven, B-3001 Leuven, Belgium; 3 Institute for Microelectronics, TU Wien, Gußhausstraße 27–29/E360, 1040 Wien, Austria; 4 Department of Mechanical Engineering, Faculty of Engineering Sciences, KU Leuven, B-3001 Leuven, Belgium

**Keywords:** interconnect, stress evolution, void nucleation, void-dynamics, copper, electromigration, microstructure, grain size

## Abstract

As electronic devices continue to shrink in size and increase in complexity, the current densities in interconnects drastically increase, intensifying the effects of electromigration (EM). This renders the understanding of EM crucial, due to its significant implications for device reliability and longevity. This paper presents a comprehensive simulation framework for the investigation of EM in nano-interconnects, with a primary focus on unravelling the influential role of microstructure, by considering the impact of diffusion heterogeneity through the metal texture and interfaces. As such, the resulting atomic flux and stress distribution within nano-interconnects could be investigated. To this end, a novel approach to generate microstructures of the conductor metal is presented, whereby a predefined statistical distribution of grain sizes obtained from experimental texture analyses can be incorporated into the presented model, making the model predictive under various scales and working conditions with no need for continuous calibration. Additionally, the study advances beyond the state-of-the-art by comprehensively simulating all stages of electromigration including stress evolution, void nucleation, and void dynamics. The model was employed to study the impact of trench dimensions on the dual damascene copper texture and its impact on electromigration aging, where the model findings were corroborated by comparing them to the available experimental findings. A nearly linear increase in normalized time to nucleation was detected as the interconnect became wider with a fixed height for aspect ratios beyond 1. However, a saturation was detected with a further increase in width for lines of aspect ratios below 1, with no effective enhancement in time to nucleation. An aspect ratio of 1 seems to maximize the EM lifetime for a fixed cross-sectional area by fostering a bamboo-like structure, where about a 2-fold of increase was estimated when going from aspect ratio 2 to 1.

## 1. Introduction

Electromigration (EM) refers to the phenomenon where the movement of atoms within a conductor is induced by a high-density electric current. This process can lead to the gradual degradation of the conductor, causing material displacement, void formation, and ultimately device failure. The variability of EM time to failure (TTF) has always been a key challenge for back-end-of-line (BEOL) reliability predictions. Experiments have shown that time to failure has a large range of values for similar interconnects fabricated by the same technology and tested under the same working conditions [1,2]. Many factors contribute to TTF variability including the variation in the actual cross-sectional area of the interconnect [3], the variation in quality of the interfaces between the interconnect’s metal and the surrounding materials, and other process related factors such as via misalignments. However, one of the most important intrinsic sources of variation is the microstructure of the interconnect. The microstructure has always been identified as a key contributor to the mechanical, thermal, transport, and electrical properties of materials [4], and not surprisingly, it has been shown to be strongly implicated in EM [5,6]. The microstructure has been identified to be the main factor causing a shortened lifetime of interconnects with downscaling of the interconnect cross-sectional area for a given current density [5], making the inclusion of microstructure in the modeling of EM in nano-interconnects a very important aspect of a comprehensive and predictive model.

Numerous scientific contributions have delved into the intricate relationship between microstructural features and the EM behavior of nanoscale interconnects. Zhang et. al. [6] conducted EM experiments on two sets of Cu-based interconnects with different average grain sizes that were realized by manipulating the parameters used during electroplating. A 2-fold increase in lifetime was observed for an average grain size of 198 nm compared to a smaller average grain size of 125 nm. A similar work studying the impact of average grain size was carried out by Choi et. al. [5], where a wider range of variation in average grain size was achieved by varying the trench width while fixing all of the other fabrication parameters, resulting in a significant enhancement in EM lifetime as the metal line width increased from the minimum to three times the minimum width. However, beyond this limit, further widening of the metal line did not yield any additional improvement in EM lifetime. Oates et al. [1] derived the diffusivity coefficients in different diffusion paths (i.e., grain boundaries and interfaces) for the estimation of drift velocity by conducting EM experiments and texture analysis on damascene interconnects fabricated with different dimensions and technological schemes.

Only a handful of studies on the numerical modeling of EM have attempted to explicitly simulate the microstructure by considering heterogeneous diffusivity domains. In contrast, to reduce model complexity and the entailed computational costs, the widely adopted approach has been homogenization using the effective diffusivity concept [7,8], where a homogenous atomic/vacancy flux through the entire interconnect’s cross-section is employed to approximate the distributed flux through the grain boundaries and interfaces. One of the few such studies was the seminal work by Gleixner [9], where the atomic flux in each type of diffusivity path was given in terms of the current density and the angle between the path and the vector of the electric current, giving a stress distribution varying between compression and tensile, and concentrated within the diffusivity paths. Later, the impact of microstructure on the void dynamics was introduced by Bower [10], where the diffusivity value along the void surface was considered to vary depending on its interaction with the grain boundaries and other interfaces, causing a variation in the atomic flux. Subsequently, the normal velocity of the void’s surface was estimated using the normal component of the volumetric atomic flux and the divergence of its tangential component. An attempt to include microstructure in both stress evolution and void dynamics was shown separately by Kteyan and Sukharev [11] using the phase field method. An innovative approach to avail of the benefits of the effective model, being its simplicity and low computational cost, while the impact of microstructure could still be approximated, was implemented [12], where the interconnect was divided into segments of polycrystalline and single crystal regions, each having its own effective diffusivity value based on the dominating diffusivity path, that is, the grain boundaries for polycrystalline regions and the cap interface for the single crystals. In essence, the incorporation of microstructure into simulations of EM is indispensable for navigating the challenges posed by the relentless march toward miniaturization in the ever-evolving landscape of nanoelectronics.

**Figure 1:**
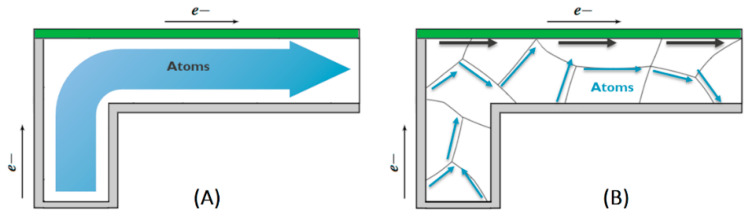
A comparison between the atomic flux model in (**A**) the homogenous effective diffusivity approach [8] and (**B**) the presented modeling approach in this work.

The model presented in this investigation constitutes a major enhancement of our antecedent model expounded comprehensively in [8], where detailed expositions and derivations for the pertinent equations and their implementation can be found. Notably, the antecedent model relied upon a singular effective diffusivity coefficient necessitating calibration for each distinct interconnect dimension and technology. In the current study, this aspect has undergone refinement, wherein the solitary effective diffusivity has been supplanted by a more precise distributed diffusivity scheme. The interconnect cross-section is treated as a heterogeneous diffusive medium with distinct paths within a generated microstructure. This approach provides a more nuanced representation of the microstructural influences on diffusive processes, capturing the impact of dimensional interconnect scaling and technological variations seamlessly. A visual comparison between the two approaches is shown in Figure 1. The presented model complements the existing literature models due to its comprehensive simulation encompassing all stages of EM including stress evolution, void nucleation, stress relaxation, and void dynamics, along with their intricate interactions with high diffusivity pathways. Additionally, its realistic microstructure generation module considers interconnect aspect ratios, demonstrating commendable alignment with the experimental observations.

The subsequent sections of this study are structured as follows. In Section 2, the implemented methodologies are described, encompassing the algorithms employed for the generation of microstructures and the methodology employed for their integration into the EM simulations. Subsequently, in Section 3, the comprehensive ability of the presented model to capture all stages of EM failure is demonstrated. The simulation framework was then used to study the impact of trench width (and thereby linewidth) on the microstructure and EM lifetime, where the simulation was compared with the experimental findings. This validation is then followed by an application of the model beyond the experimental domain to reveal the full scaling and aspect ratio impact on EM reliability. Finaly, in Section 4, a conclusive summary of the findings is provided.

## 2. Methods

**Figure 2:**
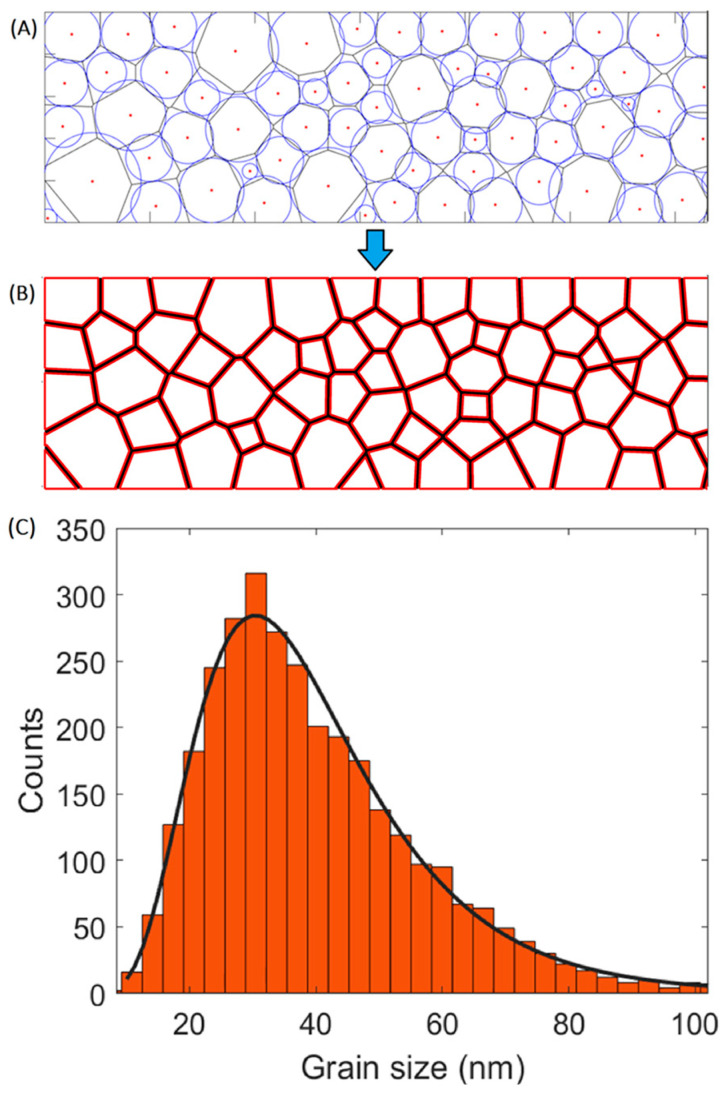
The model utilized to generate grain microstructures. (**A**) The packed circles together with their weighted Voronoi tessellation. (**B**) The created grains from the tessellation after applying the grain boundary thickness. (**C**) The grain size distribution of the generated grains (orange histogram) shown together with the lognormal grain size distribution (continuous black curve).

As a first step, a realistic grain structure has to be implemented. We proposed a 2-dimensional model, where grains span the 3rd-dimension (i.e., the linewidth). The synthesis of artificial but realistic microstructures for simulation is implemented by employing a novel methodical process. This process is based on the packing of circles of various sizes in the interconnect domain (Figure 2A), where the radii of the circles follow a prescribed statistical distribution, as shown in Figure 2C. This distribution is obtained from the experimental grain size analyses on Cu interconnects of different dimensions [5,13]. The best-fit analysis yielded a lognormal distribution of grain sizes, with an average size approximating the minimum cross-sectional dimension (i.e., the minimum of line width and height) and a standard deviation of ~0.45 × the average grain size. Initially, the simulation incorporates dynamic packing of the generated circles, which utilizes random perturbation displacement vectors with an equal probability in all directions in the 2D plane to keep the circles in continuous motion and prevent them from being fixed to a certain corner. In each iteration of the dynamic packing algorithm, a different set of perturbation displacement vectors are applied to the circles in new directions, then the model ensures contact resolution amongst the packed circles to minimize their overlap and the inter-circular empty spaces. This process continues until the sum of the distances that the circles’ centers travel during the iteration is less than 0.01 of the circles’ average radius. As such, the dynamic packing stops when the circles cannot move anymore, indicating that they have reached the best packing condition possible. An example of the dynamic packing process is provided as a video in the Supplementary Materials, and a detailed supplementary document on the dynamic packing algorithm is available online. Our algorithm stands out among other algorithms due to its unique feature of ensuring that all circular objects exist as specified from the beginning, thereby preserving the original experimental size distribution. In contrast to other algorithms, such as the random placement [14], which dynamically adds circles based on available spaces left by earlier placements, the utilized approach guarantees the inclusion of all pre-specified circle sizes. This prevents deviation of the size distribution of the circular objects during the packing process from the prescribed distribution. Subsequently, circles are used to create actual grains by employing a weighted Voronoi tessellation, also known as the power diagram [15] (Figure 2B). The circles’ centers are utilized as seeds for the Voronoi diagram, while their radii act as weights, influencing the spatial extent of each Voronoi cell to match as closely as possible with the corresponding circle [15]. This weighted Voronoi tessellation creates a microstructure that faithfully represents the specified grain size probability distribution. An example of the described steps to create a microstructure is illustrated in Figure 2.

**Figure 3:**
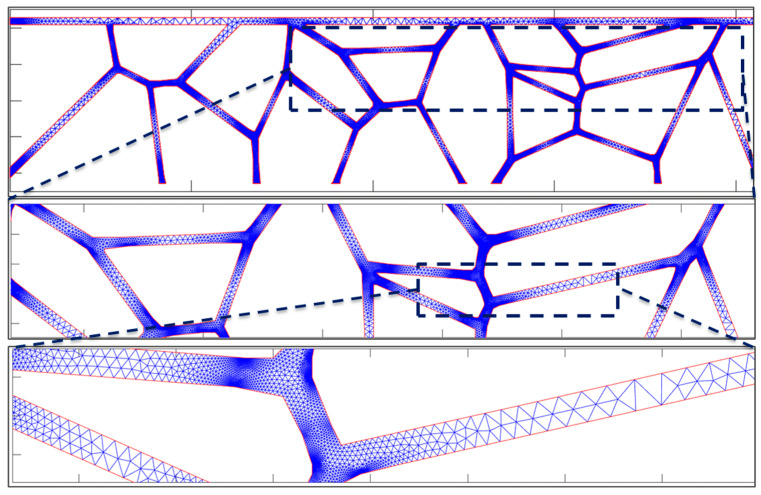
An example of the meshing scheme applied to our model. Two successive zoom-ins show how the mesh size varies from triple points to the middle of a grain boundary.

The microstructure generation module creates the computational domain devised for the resolution of atomic flux, which is a network comprising high-diffusivity paths, specifically the interconnect interface with the cap layer and the grain boundaries. Concomitantly, flux within the bulk and across metal barrier interfaces is disregarded, as substantiated by markedly lower diffusivity values (~10 order of magnitude lower for bulk) [1,16]. This geometry domain is then used in our finite element (FE) solver, employed within MATLAB^®^ (Version R2020a), to model the stress evolution with time. The numerical mesh configuration is accordingly tailored to reflect a higher density at triple points, where the divergence of the atomic flux is most pronounced, and a coarser mesh at the midpoints of the paths, as illustrated in Figure 3. For the calculation of the current density, a separate FE solver was used, following the same principles outlined in our preceding publication [8].

**Figure 4:**
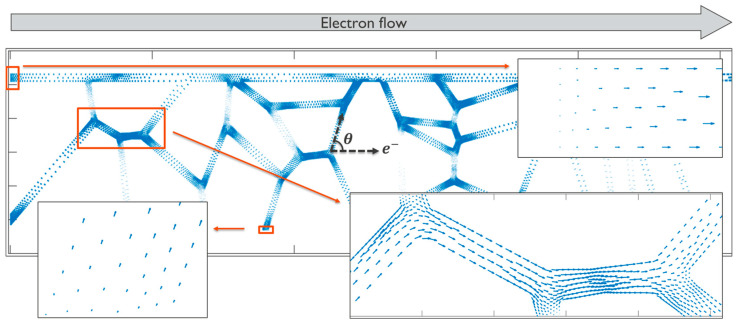
A vector plot of the atomic flux when the electron flow is from left to right. The direction of the blue arrows indicates the atomic flux direction, and their lengths indicate the flux magnitudes.

The stress evolution with time is determined by the divergence in atomic flux (i.e., depletion and accumulation of atoms) and is given by [8],

(1) $$\frac{\partial \sigma }{\partial t}=B\Omega f𝛻.J_{a}$$

where σ is the stress, t is time, B is the bulk modulus, Ω is the atomic volume, f is the vacancy relaxation volume, and $J_{a}$ is the atomic flux. The atomic flux is defined as the summation of the flux driven by the electron wind force and the flux driven by the stress gradient [9,17],

(2) $$J_{a}=\frac{D_{a}F_{e}}{\Omega K_{B}T}+\frac{D_{a}f𝛻\sigma }{K_{B}T}$$

where $D_{a}$ is the value of atomic diffusivity in the path where the atomic flux is calculated. For instance, $D_{a}=D_{gb}$ inside grain boundaries and $D_{a}=D_{cap}$ for the atomic flux of the cap interface (see Table 1). $F_{e}$ is the electron wind force, $K_{B}$ is the Boltzmann constant, and T is temperature. The electron wind force on each path is given by the amount of momentum exchange between electrons and atoms in the direction tangential to the path [9],

(3) $$F_{e}=Z^{*}e\rho j_{e}\cos\left(\theta \right)$$

where $Z^{*}$ is the effective charge, e is the electronic charge, ρ is the resistivity, $j_{e}$ is the current density, and θ is the angle that the diffusivity path of interest makes with the direction of the electric current flow, as illustrated for one of the grain boundaries in Figure 4. In this computational framework, the determination of the atomic flux within each path is initiated by employing Equations (2) and (3). A representative calculation is shown in Figure 4 for a case of electrons flowing from left to right. At the interconnect boundaries, such as at the end terminals isolated by diffusion barriers, a blocked boundary condition that ensures a zero normal component of the total flux was applied.

**Figure 5:**
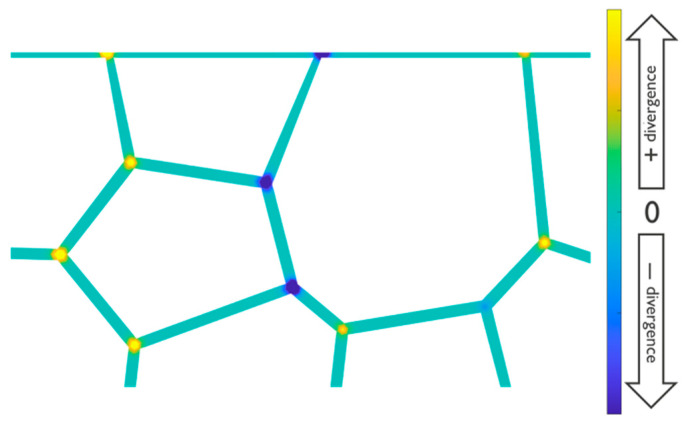
A colormap indicating the divergence of atomic flux through the diffusion path network. Divergence was non-existent along the path and had a positive or negative value at triple points depending on the type and angle of the path.

The atomic flux takes place along the fast diffusion paths, being higher when the path aligns more closely with the electron flow direction, in contrast to paths perpendicular to it. Subsequently, the flux divergence is computed, elucidating regions where atoms accumulate or deplete, notably at triple points. An illustrative calculation of flux divergence is presented in Figure 5. Finally, the evolution of stress is evaluated for each discrete time step, employing the formulation provided in Equation (1). The subsequent phases of the simulation, encompassing void nucleation and void dynamics, adhere to the methodologies expounded in our prior publication [8].

For void nucleation, it is imperative to surpass a critical tensile stress threshold, $\sigma_{crit}$, initiating void growth. Triple points within the microstructure are conducive to nucleation due to their role as focal points for flux divergence, and hence stress generation. Moreover, the lower activation energy of atoms at triple points reduces the critical stress required for voiding [18]. The chemical potential in terms of normal stress, $\sigma_{n}$, [19] is given by

(4) $$\mu =\mu_{0}-\Omega \sigma_{n}\;$$

while on the void surface, assuming negligible elastic deformation on the free surface, the chemical potential can be expressed in terms of free surface energy, γ, and curvature, k, [10] by

(5) $$\mu =\mu_{0}-\Omega \gamma k$$

where μ is the chemical potential, and $\mu_{0}$ is the stress-free potential. Utilizing the principle of continuity of chemical potential, the two expressions in Equations (4) and (5) are equal at the void surface, giving Equation (6) for the stress normal to the void’s surface, $\sigma_{s}$, as follows:

(6) $$\sigma_{s}=\gamma k$$

This stress at the void surface is introduced as a boundary condition post-nucleation. Assuming a circular initial void geometry, the initial void radius, $r_{0}$, is estimated as outlined in Equation (7) [20]. This equation was deduced by minimizing the Gibb’s free energy of a stressed continuum containing a void by variating its size.

(7) $$r_{0}=\frac{2\gamma }{\sigma_{crit}}$$

The nucleated void generates a new high diffusivity path represented by its free surface. The normal velocity of the evolving void surface under mass transport,${\;V}_{⊥}$, is determined by Equation (8) using the calculated flux on the void surface [10].

(8) $$V_{⊥}=-\delta_{s}\nabla .j_{{vol}_{//}}+j_{{vol}_{⊥}}$$

where $j_{{vol}_{//}}$ is the volumetric flux tangential to the void surface, $j_{{vol}_{⊥}}$ is the volumetric flux normal to the void surface, and $\delta_{s}$ is the void’s surface phase thickness.

**Table 1:** A list of the physical input parameters used in the simulations.

Parameter	Description	Value	Unit	Reference
B	Effective Bulk modulus	13.3	GPa	[21]
ν	Poisson ratio	0.3	-	[22]
ρ	Resistivity	50	Ohm.nm	[21]
γ	Surface free energy	1	J/m^2^	[23]
$Z^{*}$	Effective charge number	2.38	-	[21]
$j_{e}$	Current density	5	MA/cm^2^	-
T	Working temperature	100	°C	-
f	Vacancy relaxation volume	0.23	-	[24]
$\sigma_{0}$	Initial stress	185	MPa	[21]
$\sigma_{crit}$	Critical stress for voiding	400–500	MPa	[21]
$\delta_{gb}$	Grain boundary thickness	1	nm	[25,26]
$\delta_{cap}$	Cap interface thickness	0.5	nm	[25,26]
$D_{gb}$	Grain boundary diffusivity	0.95	nm^2^/s	[1]
$D_{cap}$	Cap interface diffusivity	0.56	nm^2^/s	[1]

## 3. Results and Discussion

**Figure 6:**
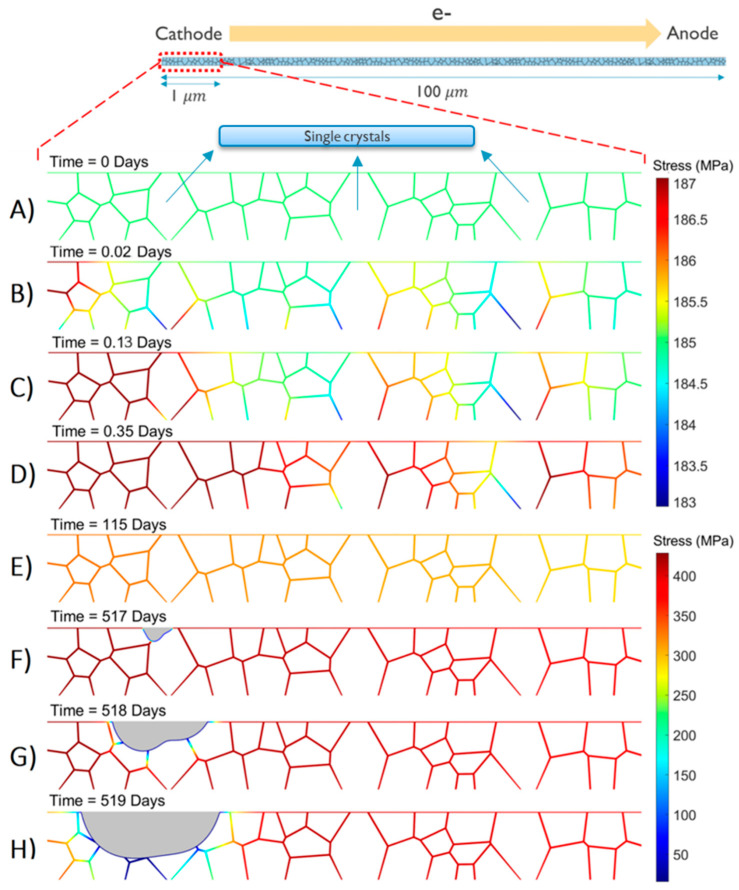
A colormap plot to show the stress and the void’s geometry change with time. Two color bars are used for clarity, where the top bar has a small range to illustrate the subtle differences in stress along the grain’s boundaries and cap interfaces during the initial stress evolution phase (**A**–**D**). The bottom bar has a wide range to illustrate the drastic stress changes pre- and post-void nucleation (**E**–**H**). Multimedia is available online.

The simulation results on stress evolution, void nucleation, and void dynamics for a 100 μm long dual damascene Cu interconnect with a linewidth of 40 nm and an aspect ratio (AR) of 2 with a conventional SiCN cap layer are shown in Figure 6, zoomed in on the region of the cathode end for clarity. See Table 1 for the physical parameters and working conditions employed in the simulations.

**Figure 7:**
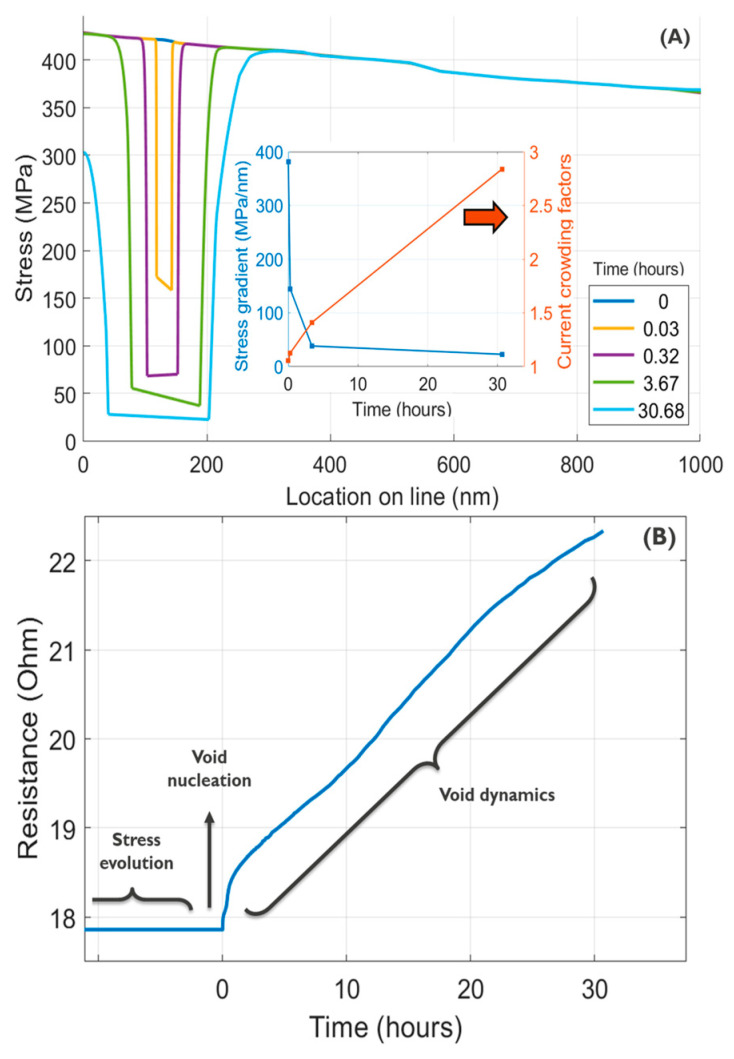
(**A**) The stress evolution along the cap interface beyond the void nucleation time. Subplot shows the stress gradient at the void surface/cap intersection point and the current crowding factor at the void surface. (**B**) The simulated electromigration induced an increase in resistance with time, showing the three stages of electromigration aging.

Because of the substantial difference in stress ranges during the initial phase of stress evolution and subsequent stages of void nucleation and dynamics, distinct color bars were employed to ensure clarity and enhance the contrast in stress visualization across all phases. A comprehensive simulation video illustrating the complete lifetime is provided in the Supplementary Materials, while selected snapshots of interest are highlighted in Figure 6. At the onset (time 0), denoted as the initiation of current flow through the interconnect, a uniform initial stress of 185 MPa was assumed, as estimated in our dedicated thermal-mechanical study [8]. This initial tensile stress originates during the cooling phase from the stress-free temperature down to the working condition temperature, attributed to the differing thermal expansion coefficients of copper (Cu) and the surrounding materials [27]. After current turn-on, momentum exchange between the electron wind and the Cu atoms induces an atomic flux through the high-diffusivity paths. Depending on the directions and magnitudes of these fluxes, flux divergence results in either atomic depletion, augmenting the initial tensile stress, or atomic accumulation, shifting the stress toward compression. Large single crystals serve as flux divergence segments, with atomic flux locally occurring solely through the cap interface. In contrast, polycrystalline segments exhibit higher flux due to the presence of both the cap interface and grain boundaries as high-diffusivity paths. Subsequently, an increasing number of atoms diffuse toward the anode end, leading to the highest tensile stress near the cathode end (see Figure 6). This stress evolution continues until reaching the minimum critical stress for voiding, assumed to occur at a triple point on the cap interface [28] (see Figure 6F). At this juncture, a void nucleates, and tensile stress locally relaxes around the nucleated void [29]. The void undergoes growth, primarily under the influence of atomic flux driven by the stress gradient, with regions intersecting high-diffusivity paths expanding more rapidly. As the void continues to grow, the stored mechanical energy is released, leading to stress relaxation and a subsequent weakening of the initially strong gradient, as depicted in Figure 7A, where the onset (time 0) is denoted in this figure as the time of void nucleation and the stress gradient is calculated at the point of intersection between the void surface and the cap interface. Simultaneously, the reduction in the interconnect’s cross-sectional area due to void growth results in an increase in interconnect resistance (see Figure 7B), generating about a 3× increase in current density around the void surface and propelling accelerated void migration toward the cathode end.

**Figure 8:**
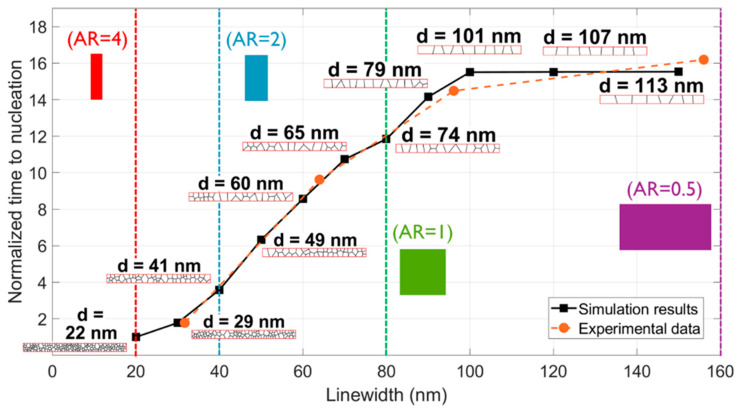
The time to nucleation normalized to the minimum value for different linewidths as estimated by our model vs. experimental results. For each simulation point, the average grain diameter, d, is shown together with a snapshot of the generated microstructure. Dashed vertical lines mark different aspect ratios. The temperature and rest of the working conditions in the simulations shown in this figure follow the reported values in [5].

Using the developed modeling framework, a comprehensive case study investigating the impact of trench width (and thereby the damascene interconnect’s linewidth) on electromigration was investigated and subsequently compared with the experimental data [5] obtained from interconnects with an identical height of 80 nm. Specifically, the experimental trials in [5] involved interconnects with linewidths of 32 nm, 64 nm, 96 nm, and 156 nm. In the simulations, the linewidth variations were systematically introduced, ranging from 20 nm to 150 nm. The rest of the simulated working conditions for the results shown in Figure 8 were adopted from [5] to match the experimental conditions. For each simulation data point, a short segment of the generated microstructure at the corresponding scale is exemplified in conjunction with the average grain size, d, in Figure 8.

It is evident that for interconnects with AR < 1, the microstructure was predominantly bamboo and transitioned toward increased polycrystallinity and smaller average grain sizes at narrower linewidths. Thereby, the increased grain boundary density increased the atomic flux and accelerated stress evolution, consequently leading to a reduced time to nucleation. The elongation in time required for nucleation grew proportionally with the expansion of line width, exhibiting a linear trend until reaching an aspect ratio of 0.8, below which it stabilized. This saturation emerged because, within this range, the constant line height acted as the limiting factor for the average grain size, leading to a saturation point in total diffusivity value and consequently the time needed for nucleation. In Figure 8, the simulation results were compared to the experimental findings in [5], demonstrating a correlation that proved the validity of the presented model.

**Figure 9:**
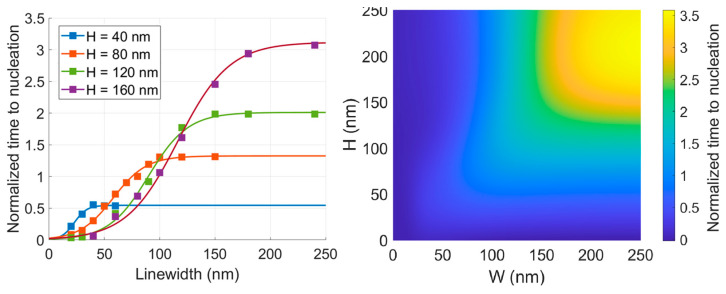
The full scaling study showing time to nucleation at various linewidth and heights. Values were normalized w.r.t the (80,80) point. All simulation points along with a sigmoid fit (**left**). Interpolated surface shown by a colormap (**right**).

Expanding the scope of this investigation to encompass various line heights provided a comprehensive understanding of the scaling effects on EM lifetime, as depicted in Figure 9. Utilizing the simulated data points (see Figure 9, left hand side plot) and using 2D interpolation, the time to nucleation was estimated for each combination of line height and width, extending up to and including 250 nm (see Figure 9, right hand side plot).

**Figure 10:**
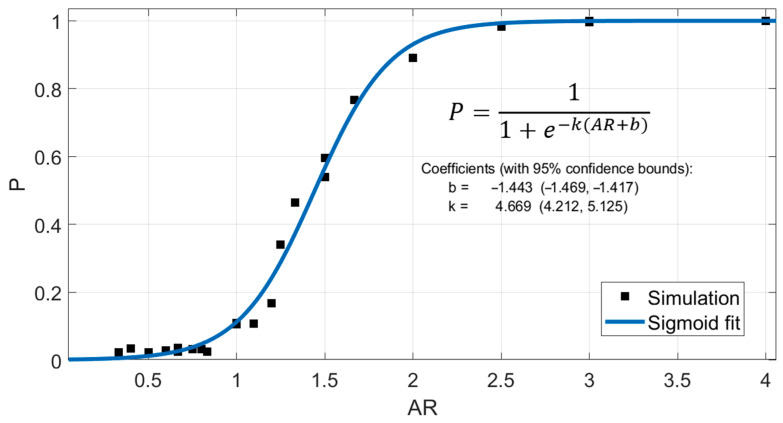
The “p” factor calculated for different microstructures, generated by our calibrated microstructure generation module for different aspect ratios (black points), along with a sigmoid fit to give an analytical formula (blue line).

The consistent trend of pronounced reliance on linewidth for high aspect ratio lines, followed by a plateauing effect, was evident across all line heights. However, due to the variation in the value of line width at which aspect ratio 1 was reached for each line height, the point of saturation varied accordingly, where doubling the line height for a fixed large line width (i.e., lying in the saturation region) was found to give about a 2.5× increase in the value of nucleation time saturation. However, for a fixed small line width (i.e., lying in the aspect ratio 1 and above region), a reduction in time to nucleation was noted as the line height increased, even if the minimum cross-sectional dimension dictating the average grain size was the fixed line width. This influence gradually reached a plateau with a further increase in line height. This phenomenon can be attributed to the probability of encountering a single crystal region or a polycrystalline region along the length of the line, quantified by the “p” factor [30], which is defined by

(9) $$P=\frac{L_{p}}{L_{p}+L_{s}}$$

where $L_{p}$ and $L_{s}$ are the mean length of the polycrystalline and single crystal regions, respectively. In a single crystal region, the atomic flux relies solely on the cap interface as the primary high diffusivity pathway. Consequently, even with a fixed average grain size dictated by the minimum cross-sectional dimension, elevating the line height diminishes the likelihood of single crystal regions to persist (indicated by a higher “p” factor), until such regions become absent for high aspect ratios (resulting in the “p” factor reaching a maximum of 1). Consequently, subsequent increases in line height exert a minimal influence on the time to nucleation. The “p” factor as a function of aspect ratio (AR) is shown in Figure 10, assuming that the quality of interfaces is the same across different scales. The results from our simulations in Figure 10 were in perfect agreement with the reported experimental studies in [12] for the “p” factor measured at aspect ratios 2 and 3.

**Figure 11:**
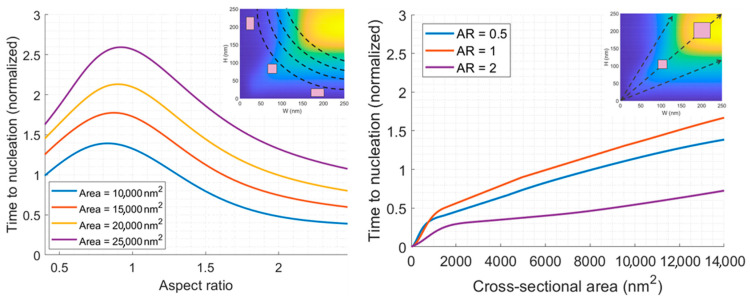
Time to nucleation (normalized) vs. aspect ratio at different cross-sectional areas (**left**). Time to nucleation (normalized) vs. cross-sectional area at different aspect ratios (**right**). For each figure, an inset is given to show the lines of interest on the full scaling colormap.

In addition to the previously discussed cases of varying line height while keeping width constant and vice versa, represented by vertical and horizontal lines on the full scaling colormap (Figure 9, right), there were two additional lines of interest. These included iso-volumetric lines, indicating a fixed cross-sectional area with different aspect ratios (Figure 11, left), and fixed aspect ratio scaling lines, indicating different cross-sectional areas (Figure 11, right). The results indicate that an aspect ratio of 1 maximizes the EM lifetime for each cross-sectional area, with an estimated 2-fold increase when moving from an aspect ratio of 2 to 1. This can be explained by the fact that at an aspect ratio of 1, both the line width and height are the minimum dimension, allowing for microstructure grains to fill the entire interconnect trench cross-section. This fosters a bamboo-like structure, reduces the probability of polycrystalline regions, creates a low diffusivity medium for atomic flux, and extends the EM lifetime.

**Figure 12:**
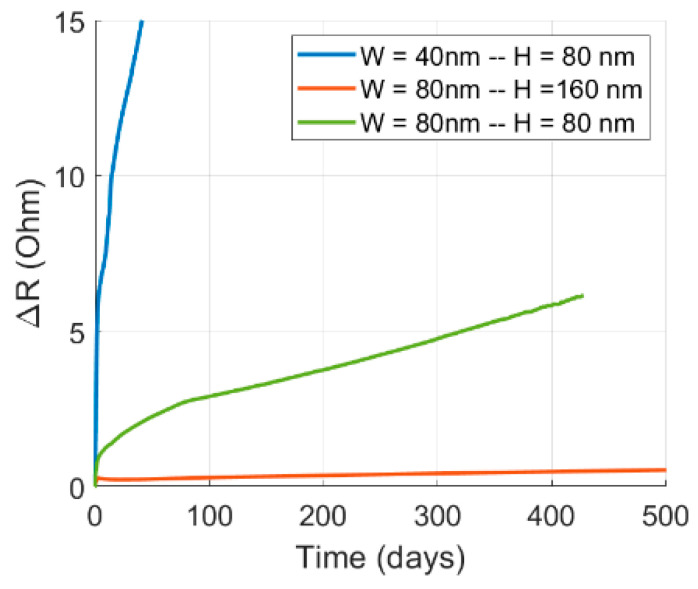
The EM-induced increase in resistance above the initial value as a function of time for different cross-sectional dimensions. Time 0 was defined as the time of void nucleation for each case.

Examining the escalation of resistance post-void nucleation across various dimensions provides insights into the influence of scaling on void growth, as depicted in Figure 12. The figure illustrates three distinct combinations of line height and width. Notably, time 0 was defined as the moment of void nucleation for each case, acknowledging the varying times to nucleation for each case, as highlighted earlier.

A comparison of the scenario where both the line height and width were 80 nm to the case where the line height was 80 nm, and the width was 40 nm illustrated the consequence of altering the line width while maintaining a constant line height. Doubling the line width resulted in an approximately 200-fold increase in the time required to achieve the same rise in resistance. This disparity arose from the heightened presence of grain boundaries interacting with the void surface, attributable to a smaller average grain size in the case of a smaller width. Consequently, a larger flux of atoms migrated away from the void surface, accelerating its growth. Likewise, comparing the scenario where both the line height and width were 80 nm to the case where the line height was 160 nm, and the width was 80 nm elucidated the effect of varying the line height while keeping the line width constant. Doubling the line height led to an approximately 500-fold increase in the time required to attain the same rise in resistance. This discrepancy was attributed to the larger cross-sectional area, necessitating a greater distance for the void surface to traverse in order to generate an equivalent resistive impact in the case of a larger height. Finally, comparing the scenario where the line height was 80 nm, and the width was 40 nm with the case where the line height was 160 nm, and the width was 80 nm showed the impact of scaling both dimensions while maintaining a constant aspect ratio. This comparison highlights the most pronounced variation in the time needed for the void to generate the same resistive impact, attributable to the combined influence of a larger average grain size and a larger cross-sectional area.

## 4. Conclusions

In conclusion, our study presented a comprehensive simulation framework for assessing electromigration failure in nano-interconnects, wherein the nuanced impacts of microstructure and linewidth scaling are systematically examined. Our simulation framework captured the entire spectrum of EM stages including stress evolution, void nucleation, and void dynamics. The modeling of stress evolution unveiled the intricate interplay of atomic flux, stress gradients, and microstructural features, shedding light on the dynamics leading to void nucleation. The subsequent void growth phase was characterized by complex interactions including the release of stored mechanical energy and changes in stress gradients, influencing the void growth rates and interconnect resistance. The presented model, due to its fully physics-based foundations and texture-level considerations, is well-suited for simulating electromigration in any crystalline metal, provided the corresponding physical parameters of the material are used in the simulation. According to our model’s predictions for Cu interconnects, under the assumption of consistent interface quality across various scales and for the same cross-sectional area, the proportion of polycrystalline segments within the interconnect will substantially rise from approximately 10% to around 90% of the total length, causing a reduction in lifetime as the aspect ratio escalates from 1 to 2. Notably, for a given cross-sectional area, an aspect ratio of 1 optimizes the EM lifetime by minimizing the grain boundary density and fostering a bamboo-like structure.

## Data Availability

The data supporting the findings of this study are available within the article and the Supplementary Materials.

## Supplementary Materials

The following supporting information can be downloaded at: https://www.mdpi.com/article/10.3390/nano14221834/s1. Detailed explanation of the developed “Dynamic Packing” algorithm mentioned in this work. Figure S1: Grain size distribution is used to generate circles; Figure S2: Initial locations of generated circles randomly scattered within the polygon; Figure S3: polygon boundary contact resolution displacement; Figure S4: inter-circular contact resolution displacement; Figure S5: Minimization curves for overlap area between circles and the empty gap area as simulation progresses; Video S1: An example of the dynamic packing process; Video S2: A comprehensive simulation video illustrating the results shown in Figure 6 in this work showing the stress and the void’s geometry change with time.
