# Supplementary material for: A Comprehensive Microstructure-Aware Electromigration Modeling Framework; Investigation of the Impact of Trench Dimensions in Damascene Copper Interconnects
Source: Nanomaterials (Basel). 2024 Nov 16;14(22):1834. doi: 10.3390/nano14221834 (PMC11597653; doi:10.3390/nano14221834)
Supplement: Supplementary file 1 [file nanomaterials-14-01834-s001.zip › Supplemntary Information_Dynamic packing.pdf]

Supplementary information on “Dynamic Packing” algorithm

A Comprehensive Microstructure-Aware Electromigration Modeling Framework; Investigation of the Impact of Trench Dimensions in Damascene Copper Interconnects

Ahmed Sobhi Saleh, Kristof Croes, Hajdin Ceric, Ingrid De Wolf and Houman Zahedmanesh

Ahmed Sobhi Saleh, Kristof Croes, Hajdin Ceric, Ingrid De Wolf and Houman Zahedmanesh
Correspondence: houman.zahedmanesh@imec.be

Dynamic packing algorithm

Problem definition: Given a two-dimensional polygon with a finite area A and S sides, determine the optimal arrangement of a set of circles with varying radii, distributed according to a probability distribution P, on the polygon's surface. The objective is to minimize both the overlap between circles and the empty gaps between them.

Algorithm steps:

1) Generate circles:

The specified probability distribution for grain size is employed to generate circles by treating the grain size as the diameter of a circle (see Fig. 1). Circles are generated iteratively from the distribution using a Monte-Carlo approach, until the sum of their areas closely approximates the area of the boundary polygon, as described in Eq. (1).

$$\sum_{i=1}^{N} A_i \cong A \qquad (1)$$

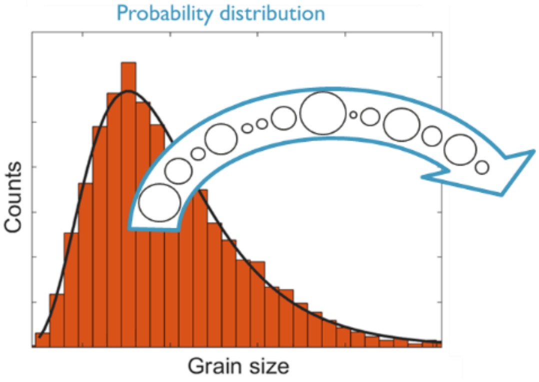

Supplementary Figure S1: Grain size distribution is used to generate circles

2) Give initial locations:

The centers of the generated circles are randomly distributed within the boundary polygon, with each circle having an equal probability of being positioned at any location inside the polygon, as illustrated in Fig. 2.

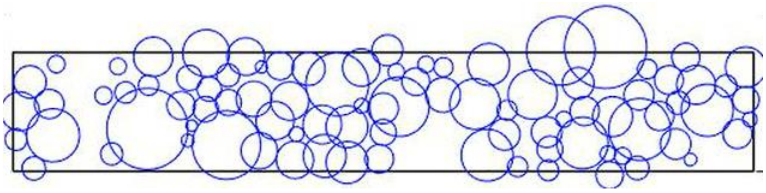

Supplementary Figure S2: Initial locations of generated circles randomly scattered within the polygon

3) Apply perturbation:

To prevent the circles from becoming stuck in specific locations and to ensure they reach the true stable final configuration, continuous motion is enforced by applying a perturbation displacement vector during each location update iteration. The magnitude of this displacement is identical for all circles and is defined in Eq. (2), such that the total displacement sum is half of the convergence criterion, ϵ, ensuring that perturbations do not inhibit convergence. Here, ϵ is chosen to be 0.01 of the average circles' radius. The displacement direction varies for each circle, with all angles in the 2D plane having equal probability.

$$\left|\vec{D}_{i_{perturb}}\right| = \frac{\epsilon}{2N} \qquad (2)$$

The new positions of the circle centers are then obtained by adding the corresponding perturbation displacement vector to their previous positions, as described in Eq. (3).

$$\overrightarrow{loc}_{i_{per}} = \overrightarrow{loc}_i + \vec{D}_{i_{perturb}} \qquad (3)$$

4) Apply contact resolution displacements:

Two displacement vectors are computed and applied to each circle to resolve contacts. The first vector addresses contact with the polygon boundaries. The magnitude of this displacement vector is determined according to Eq. (4), where *sm* is a smoothing factor, set to 0.9, to reduce abrupt changes in circle positions and thereby facilitate faster convergence. The term k_s represents the overlap distance between the circle and the s-th boundary of the polygon, as depicted in Fig. 3. The direction of the displacement vector follows the normal to the boundary, pointing inward toward the polygon.

$$\left|\vec{D}_{is_{boundary}}\right| = sm \times k_s \qquad (4)$$

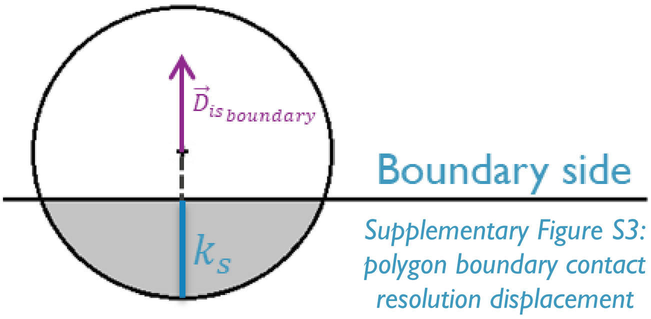

Supplementary Figure S3: polygon boundary contact resolution displacement

The second displacement vector resolves contact between circles. Its magnitude is computed as shown in Eq. (5), where h_{ij} represents the overlap between two circles i and j, as illustrated in Fig. 4. Half of this overlap distance is applied to each circle, scaled by the smoothing factor *sm*. The displacement is directed along the line connecting the centers of the two circles, pulling them away from each other.

$$\left|\vec{D}_{ij_{overlap}}\right| = sm \times \frac{h_{ij}}{2} \qquad (5)$$

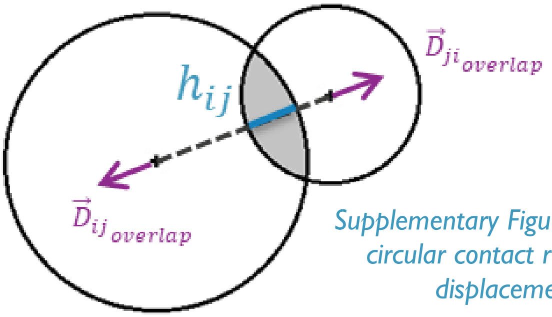

Supplementary Figure S4: inter-circular contact resolution displacement

The center of each circle is then updated by applying the total displacement, which is the sum of all resolution vectors. This process is described in Eqs. (6) and (7), ensuring that each circle's position is adjusted according to the combined effects of boundary and circle contact resolution.

$$\vec{D}_i = \sum_{s=1}^{S} \vec{D}_{is_{boundary}} + \sum_{j=1}^{N-1} D_{ij_{overlap}}, \qquad i \neq j \qquad (6)$$

$$\overrightarrow{loc}_{i_{new}} = \overrightarrow{loc}_{i_{per}} + \vec{D}_i \qquad (7)$$

5) Repeat step 3 and 4 until convergence:

Steps 3 and 4 are repeated in an iterative process, updating the circle centers at each iteration. During each iteration, the total displacement of the circle centers is calculated and compared to the convergence threshold ϵ. The simulation terminates once the total displacement falls below this threshold, as described in Eq. (8).

$$\sum_{i=1}^{N} |\overrightarrow{loc}_{i_{new}} - \overrightarrow{loc}_i| < \epsilon \qquad (8)$$

An examples illustrating how dynamic packing minimizes both the overlap area between circles and the empty gap area are shown in Fig. 5. The algorithm efficiently achieves near-minimal values for both areas within a few iterations. Additionally, a stabilization phase, where no significant further minimization occurs, is observed prior to convergence.

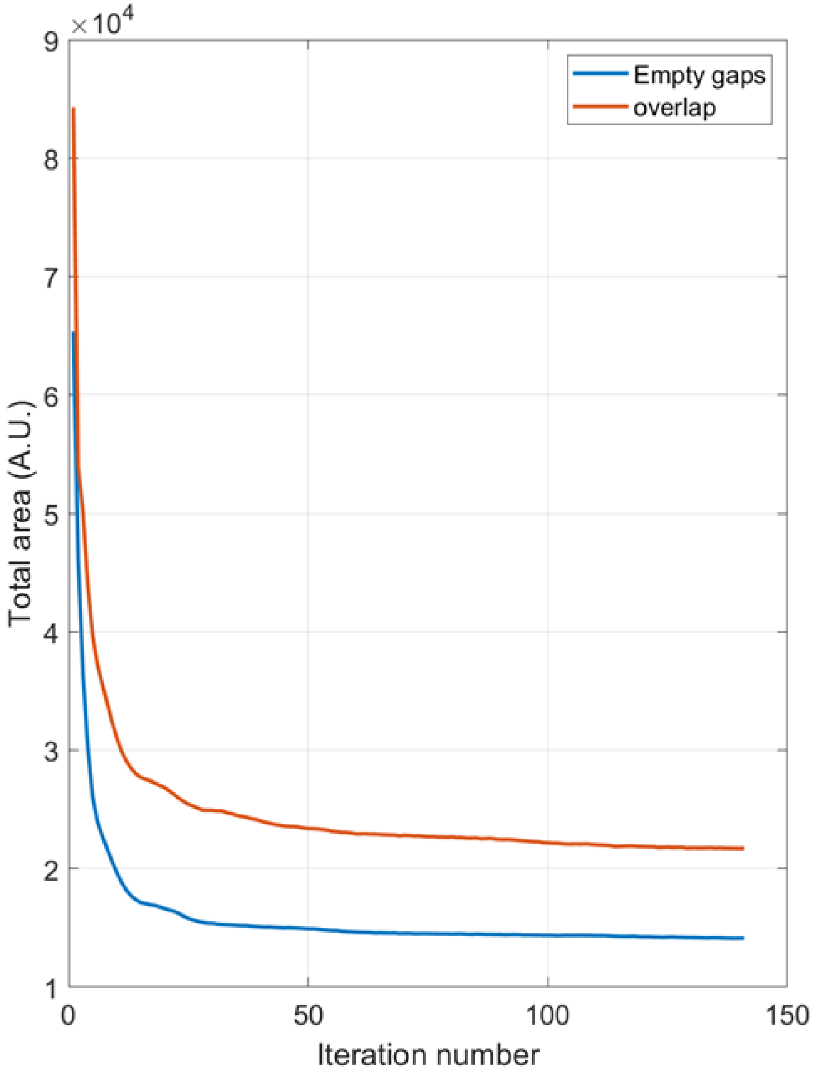

Supplementary Figure S5: Minimization curves for overlap area between circles and the empty gap area as simulation progresses.
